# Identification of Sarcopenic Obesity by Fat‐to‐Muscle Ratio in Older Adults: A Cohort Study

**DOI:** 10.1002/jcsm.70174

**Published:** 2026-01-20

**Authors:** Daisuke Kakita, Kenji Harada, Satoshi Kurita, Masanori Morikawa, Chiharu Nishijima, Kazuya Fujii, Hiroyuki Shimada, Hidenori Arai

**Affiliations:** ^1^ Department of Preventive Gerontology, Center for Gerontology and Social Science National Center for Geriatrics and Gerontology Obu Aichi Japan; ^2^ Medical Science Division, Department of Medical Sciences, Graduate School of Medicine, Science and Technology Shinshu University Matsumoto Nagano Japan; ^3^ Department of Frailty Research, Center for Gerontology and Social Science National Center for Geriatrics and Gerontology Obu Aichi Japan; ^4^ Japan Society for the Promotion of Science Tokyo Japan; ^5^ Cognitive Function Research, Aging Research (Partnership field) Nagoya University Graduate School of Medicine Nagoya Aichi Japan; ^6^ National Centre for Geriatrics and Gerontology Obu Aichi Japan

**Keywords:** fat‐to‐muscle ratio, preventive healthcare, sarcopenic obesity, screening tool

## Abstract

**Background:**

The diagnosis of sarcopenic obesity has been established in Europe and Japan, but screening tools remain inconsistent and lack standardization. The fat‐to‐muscle ratio (FMR) is a potential screening measure for sarcopenic obesity; however, its diagnostic accuracy compared with other tools has not been evaluated. This study compared the diagnostic performance of several screening tools for sarcopenic obesity.

**Methods:**

This cross‐sectional analysis used data from the National Center for Geriatrics and Gerontology Study of Geriatric Syndromes (NCGG‐SGS), a national cohort study conducted in Japan. In total, 7916 community‐dwelling older adults (mean ± standard deviation age 73.5 ± 6.2 years, 54.8% females) were included. Sarcopenic obesity was diagnosed by the Japanese Working Group on Sarcopenic Obesity (JWGSO) criteria. The FMR and phase angle (PhA) were measured using the bioelectrical impedance analysis (BIA).

**Results:**

Logistic regression analysis indicated that most screening tools, treated as continuous variables, were independently associated with sarcopenic obesity after adjustment for covariates; FMR (female: per 1‐SD odds ratio [OR] = 3.06, 95% confidence interval [CI] = 2.53–3.71; male: OR = 3.09, 95% CI = 2.67–3.58), BMI (female: OR = 2.85, 95% CI = 2.35–3.58; male: OR = 1.43, 95% CI = 1.26–1.62), waist circumference (WC) (female: OR = 2.26, 95% CI = 1.86–2.74; male: OR = 1.24, 95% CI = 1.08–1.41) and PhA (female: OR = 1.12, 95% CI = 0.93–1.34; male: OR = 0.65, 95% CI = 0.56–0.76). Receiver operating characteristic (ROC) analysis showed moderate predictive ability for each screening tool: FMR (female: area under the curve [AUC] = 0.82, 95% CI = 0.79–0.85; male: AUC = 0.81, 95% CI = 0.79–0.84), BMI (female: AUC = 0.76, 95% CI = 0.72–0.79; male: AUC = 0.55, 95% CI = 0.51–0.59), WC (female: AUC = 0.70, 95% CI = 0.66–0.75; male: AUC = 0.53, 95% CI = 0.49–0.57) and PhA (female: AUC = 0.63, 95% CI = 0.58–0.69; male: AUC = 0.70, 95% CI = 0.67–0.74).

**Conclusions:**

The findings suggest that the FMR is a more effective screening tool for identifying sarcopenic obesity than BMI, WC or PhA among community‐dwelling older adults. Longitudinal studies are needed to confirm its predictive value across broader populations.

## 1 Introduction

Sarcopenic obesity, defined as the coexistence of sarcopenia and obesity, is characterized by reduced muscle mass and impaired muscle function [1, 2]. Its prevalence is rising with population ageing [3] and has been linked to adverse health outcomes [4], including reduced quality of life [5], higher risk of falls [6] and worsening metabolic disorders [7]. It is also associated with greater risks of mortality [8] and disability [9] than sarcopenia alone, underscoring the importance of early identification and intervention.

The diagnosis of sarcopenic obesity has been established in Europe by the European Society for Clinical Nutrition and Metabolism [10] and the European Association for the Study of Obesity (EASO) [11]. In Japan, the Japanese Working Group on Sarcopenic Obesity (JWGSO) developed the diagnostic criteria in 2024 [12]; however, these criteria have not yet been validated in Asian populations. The diagnostic standards and screening tools for sarcopenic obesity require global standardization. Because of sex‐related differences in body composition, the cutoff points used to define sarcopenic obesity vary between males and females, although the diagnostic framework, low muscle mass, low muscle strength and excess adiposity remain consistent across sexes.

The European and Japanese diagnostic criteria for sarcopenic obesity recommend body mass index (BMI) and waist circumference (WC) as screening tools; however, BMI and WC do not account for variation in muscle mass and may therefore be unsuitable for identifying sarcopenic obesity [13, 14]. Phase angle (PhA), a bioelectrical impedance‐derived indicator, is strongly associated with sarcopenia [15] and may also serve as a screening tool, although its relationship with sarcopenic obesity remains unclear.

Recently, the fat‐to‐muscle ratio (FMR), the ratio of fat mass to muscle mass, has been proposed as another potential screening measure for sarcopenic obesity [16]. FMR can be calculated using data from a body composition analyser by dividing fat mass by muscle mass. Prior studies have shown that FMR and similar indices, such as the muscle‐to‐fat ratio, are associated with cardiometabolic risk [16], dementia [17], fracture [16] and mortality [18]. Our previous research also demonstrated that higher FMR was linked to a greater risk of disability after 5 years [19]. However, no studies have directly examined the association between FMR and sarcopenic obesity or compared its screening utility with that of other tools. Therefore, this study aimed to compare FMR with other screening tools for sarcopenic obesity and determine whether FMR shows a stronger association with sarcopenic obesity than existing measures.

## 2 Methods

### 2.1 Study Design and Participants

**FIGURE 1:**
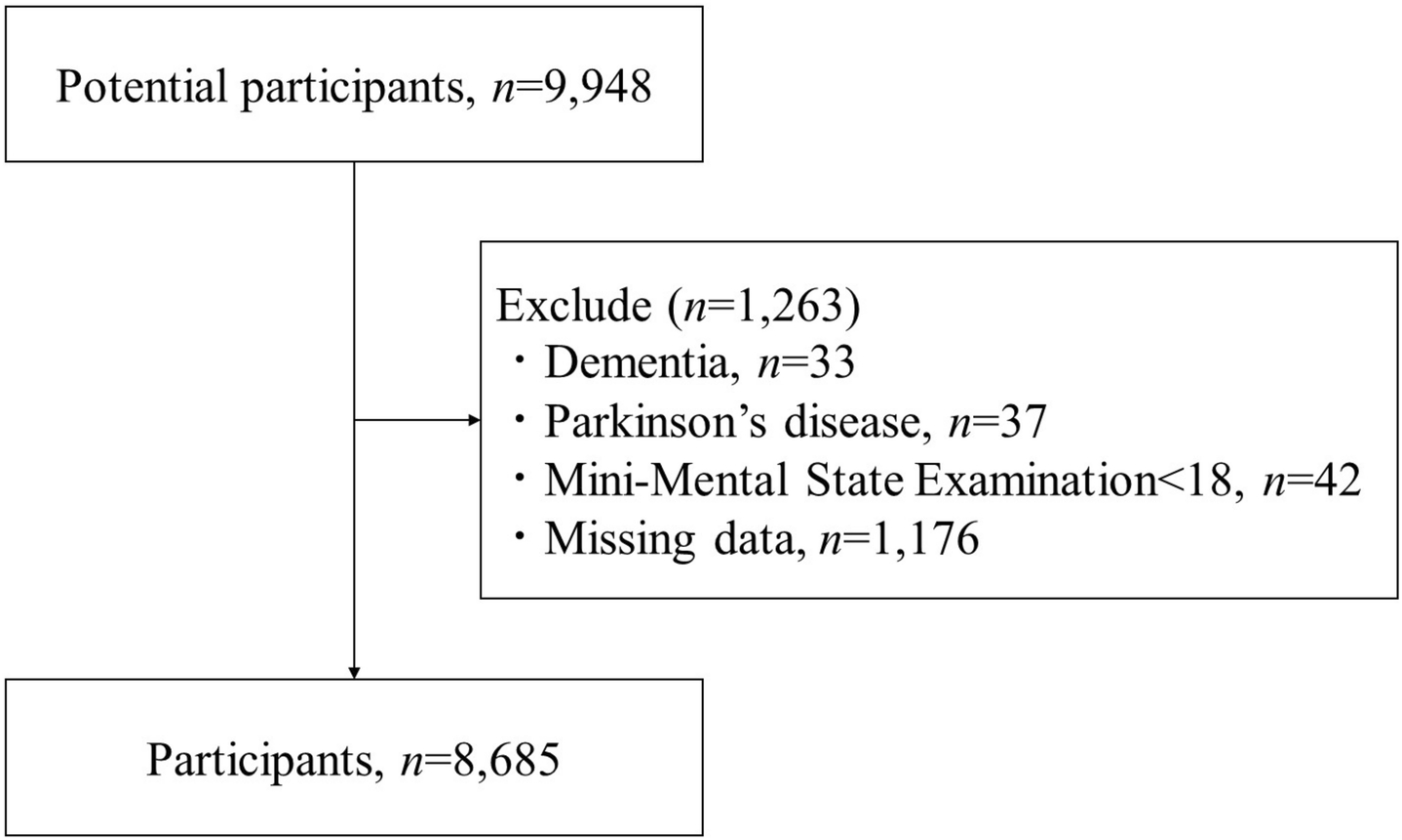
Process flow for data inclusion.

This cross‐sectional study used data from the National Center for Geriatrics and Gerontology Study of Geriatric Syndromes (NCGG‐SGS), a community‐based cohort conducted in Japan [20, 21]. The NCGG‐SGS established a screening programme for geriatric syndromes and evaluated evidence‐based interventions for their prevention [20]. Community‐dwelling adults aged 60 or older were recruited from Obu, Nagoya and Takahama between 2013 and 2016 [22]. Participants with a history of dementia (*n* = 33), Parkinson's disease (*n* = 37), a Mini‐Mental State Examination (MMSE) score [23] below 18 (*n* = 42) or missing data (*n* = 1176) were excluded. A total of 8685 participants (mean ± standard deviation age 73.6 ± 6.2 years, 54.6% female) were included in the analysis (Figure 1). The study protocol was approved by the Ethics Committee of the NCGG (Registration Number: 1440‐8), and informed consent was obtained from all participants before enrolment.

### 2.2 Assessment of Sarcopenic Obesity

Sarcopenic obesity was diagnosed according to the criteria of the JWGSO [12]. Handgrip strength and appendicular skeletal muscle mass (ASM) adjusted for BMI were used to assess sarcopenia, whereas body fat percentage and visceral fat area (VFA) were used to assess obesity.

Handgrip strength was measured twice on each side using a digital dynamometer (TKK 5101 Grip‐D; Takei, Tokyo, Japan) following a standardized protocol. Maximal isometric contractions of the hands were sustained for 3 s each and repeated twice with the participant in a standing position (or seated if unable to stand unassisted) and full elbow extension. The highest value was used for analysis. Low muscle strength was defined according to the JWGSO criteria, with cutoff values of < 28 kg for men and < 18 kg for women.

All body composition indices except VFA were measured using bioelectrical impedance analysis (BIA) (MC‐980A; Tanita). Low muscle mass was defined using JWGSO diagnostic thresholds: ASM < 0.789 kg/BMI in men and < 0.512 kg/BMI in women. Obesity was defined as a body fat percentage ≥ 20% in men and ≥ 30% in women, or a VFA ≥ 100 cm^2^ in either sex, according to established criteria [12]. VFA was measured using abdominal BIA (EW‐FA90; Panasonic), an authorized medical device in Japan (No. 22500BZX00522000). The EW‐FA90 is a portable instrument that enables efficient measurement of VFA. A measuring belt and an electric pad were attached to each participant's abdomen, and all measurements were taken in the standing position. This method is based on differences in electrical resistance between fat and other tissues: Intra‐abdominal fat has higher electrical resistance, and its tension correlates with VFA. VFA was calculated from voltage and WC. The bioelectrical impedance method shows a strong correlation with VFA determined by abdominal computed tomography (*r* = 0.88), exceeding that of WC or BMI alone [19]. This approach was therefore considered valid. Because of device limitations, the minimum measurable VFA was 10 cm^2^, and smaller values were rounded to 10 cm^2^. Sarcopenic obesity was diagnosed when participants met all three conditions: low muscle mass, low muscle strength and obesity.

### 2.3 Screening Tools for Sarcopenic Obesity

According to the recommendations of JWGSO [12], BMI and WC were included as key anthropometric indicators for screening sarcopenic obesity. In this study, BMI was calculated as weight (kg) divided by height squared (m^2^), and WC was measured at the umbilical level in the standing position using the measuring belt attached to the body composition analyser (EW‐FA90; Panasonic), which also functions as a tape measure.

Fat and muscle mass were assessed using a multifrequency BIA (MC‐980A, Tanita Corp., Tokyo, Japan) to evaluate whole‐body and segmental composition. The hand electrodes were held in contact with all five fingers, and the heels and forefeet were positioned on circular foot electrodes. During measurement, participants were instructed to slightly abduct their arms and legs to avoid contact between limbs and electrodes. FMR was calculated as total fat mass divided by total muscle mass [19]. BIA measures whole‐body impedance (*Z*), which represents the opposition of body tissues to alternating electrical current and consists of two components: resistance (*R*) and reactance (*Xc*), expressed as *Z* ^2^ = *R* ^2^ + *Xc* ^2^. PhA was derived from the arctangent of the ratio of *Xc* to *R* and used as a screening parameter for sarcopenia.

### 2.4 Potential Confounding Factors

Age, sex, living arrangement (living alone or cohabiting), smoking status, years of education, number of medications, medical history (heart disease, diabetes, depression, cancer and osteoporosis) and cognitive function were included as covariates. Data on living arrangements, smoking, educational history, medication use and comorbidities were collected through face‐to‐face interviews. Living arrangement was assessed by asking, ‘Do you currently live alone?’ Smoking status was assessed by asking, ‘Do you currently smoke cigarettes?’ and categorized as current or former/never smoker [24]. Cognitive function was evaluated using the MMSE [23].

## 3 Statistical Analysis

Baseline characteristics of the nonsarcopenic obesity and sarcopenic obesity groups were compared by sex using independent *t* tests for continuous variables and chi‐square tests for categorical variables. The diagnostic accuracy of each screening tool for sarcopenic obesity was evaluated using receiver operating characteristic (ROC) analysis, with the area under the curve (AUC) as an indicator of discriminative ability. Differences between AUCs were compared using the DeLong test to assess statistical significance between screening tools. Sensitivity, specificity, positive predictive value (PPV), negative predictive value (NPV) and overall diagnostic accuracy were calculated using both the JWGSO‐defined cutoffs and those derived from this study. The optimal cutoff point for each screening tool was identified using the Youden index, defined as (sensitivity + specificity − 1) [25]. Logistic regression analysis was used to estimate standardized (per 1SD) odds ratios (ORs) and 95% confidence intervals (CIs) for associations between each screening measure and sarcopenic obesity. Because of known sex‐related differences in body composition, all analyses were stratified by sex. Additional subgroup analyses were conducted among participants aged ≤ 75 years, corresponding to the age range specified in the JWGSO criteria. All statistical analyses were performed using Stata software (Version 18.0; MP Parallel Edition, Stata Corp LLC, College Station, TX, United States). A *p* ‐value of < 0.05 was considered statistically significant.

## 4 Results

### 4.1 Participants' Characteristics

The characteristics of participants in the nonsarcopenic and sarcopenic obesity groups, as defined according to the JWGSO criteria and stratified by sex, are summarized in Table 1. Among females, significant differences were observed in age, years of education, number of medications, physical inactivity, living arrangement, osteoporosis, osteoarthritis, GDS score and MMSE score. Among males, significant differences were also noted in smoking status and hypertension, whereas living arrangements did not differ significantly between groups. All screening tools showed significant differences between nonsarcopenic and sarcopenic obesity groups in both sexes.

**TABLE 1:** Characteristics of sarcopenic obesity by sex.

	Female (*n* = 4745)			Male (*n* = 3940)		
	Nonsarcopenic obesity	Sarcopenic obesity		Nonsarcopenic obesity	Sarcopenic obesity	
	*n* = 4624	*n* = 121	*p* value	*n* = 3694	*n* = 246	*p* value
Age, years	73.3 (6.1)	80.8 (5.6)	< 0.001	73.3 (6.0)	77.7 (7.4)	< 0.001
Education, years	11.3 (2.2)	10.1 (2.2)	< 0.001	12.3 (2.8)	10.9 (2.8)	< 0.001
Medication, number	3.1 (2.7)	4.2 (3.0)	< 0.001	2.9 (2.7)	3.7 (3.0)	< 0.001
Physical inacitivity, yes	3473 (75.1%)	116 (95.9%)	< 0.001	2643 (71.5%)	204 (82.9%)	< 0.001
Living alone, yes	838 (18.1%)	35 (28.9%)	0.002	264 (7.1%)	19 (7.7%)	0.73
Current smoking, yes	105 (2.3%)	1 (0.8%)	0.56	563 (15.2%)	40 (16.3%)	0.002
Hypertension, yes	2112 (45.7%)	65 (53.7%)	0.08	1771 (47.9%)	140 (56.9%)	0.006
Heart disease, yes	607 (13.1%)	21 (17.4%)	0.18	631 (17.1%)	36 (14.6%)	0.32
Osteoporosis, yes	979 (21.2%)	43 (35.5%)	< 0.001	58 (1.6%)	12 (4.9%)	< 0.001
Respiratory disease, yes	562 (12.2%)	17 (14.0%)	0.53	557 (15.1%)	35 (14.2%)	0.72
Osteoarthritis, yes	1215 (26.3%)	49 (40.5%)	< 0.001	459 (12.4%)	41 (16.7%)	0.053
Diabetes, yes	491 (10.6%)	19 (15.7%)	0.075	591 (16.0%)	45 (18.3%)	0.34
Geriatric depression scale	2.9 (2.6)	3.7 (2.8)	0.001	2.8 (2.8)	4.0 (3.2)	< 0.001
Minimental state examination	26.8 (2.5)	25.2 (2.9)	< 0.001	26.3 (2.6)	25.3 (3.0)	< 0.001
Appendicular skeletal muscle mass/BMI	0.624 (0.076)	0.479 (0.027)	< 0.001	0.893 (0.091)	0.712 (0.074)	< 0.001
Grip strength, kg	21.9 (4.1)	15.1 (2.5)	< 0.001	33.8 (5.8)	23.4 (3.5)	< 0.001
Body fat percentage, %	32.4 (6.9)	40.4 (5.4)	< 0.001	22.7 (5.5)	29.5 (5.9)	< 0.001
Visceral fat area, cm^2^	65.4 (30.9)	89.2 (33.1)	< 0.001	96.6 (44.4)	107.2 (44.2)	< 0.001
Body mass Index, kg/m^2^	22.9 (3.3)	26.0 (3.5)	< 0.001	23.5 (2.9)	24.3 (4.2)	< 0.001
Waist circumference, cm	82.3 (9.2)	89.0 (8.9)	< 0.001	86.1 (8.3)	87.1 (8.3)	0.062
Fat to muscle ratio	0.5 (0.2)	0.7 (0.2)	< 0.001	0.3 (0.1)	0.5 (0.2)	< 0.001
Phase angle, rad	4.6 (0.5)	4.4 (0.6)	< 0.001	5.2 (0.6)	4.8 (0.6)	< 0.001

Abbreviation: BMI, body mass index.

### 4.2 Sex‐Specific Cutoff Points and Accuracy of Screening Tools Against Sarcopenic Obesity

**FIGURE 2:**
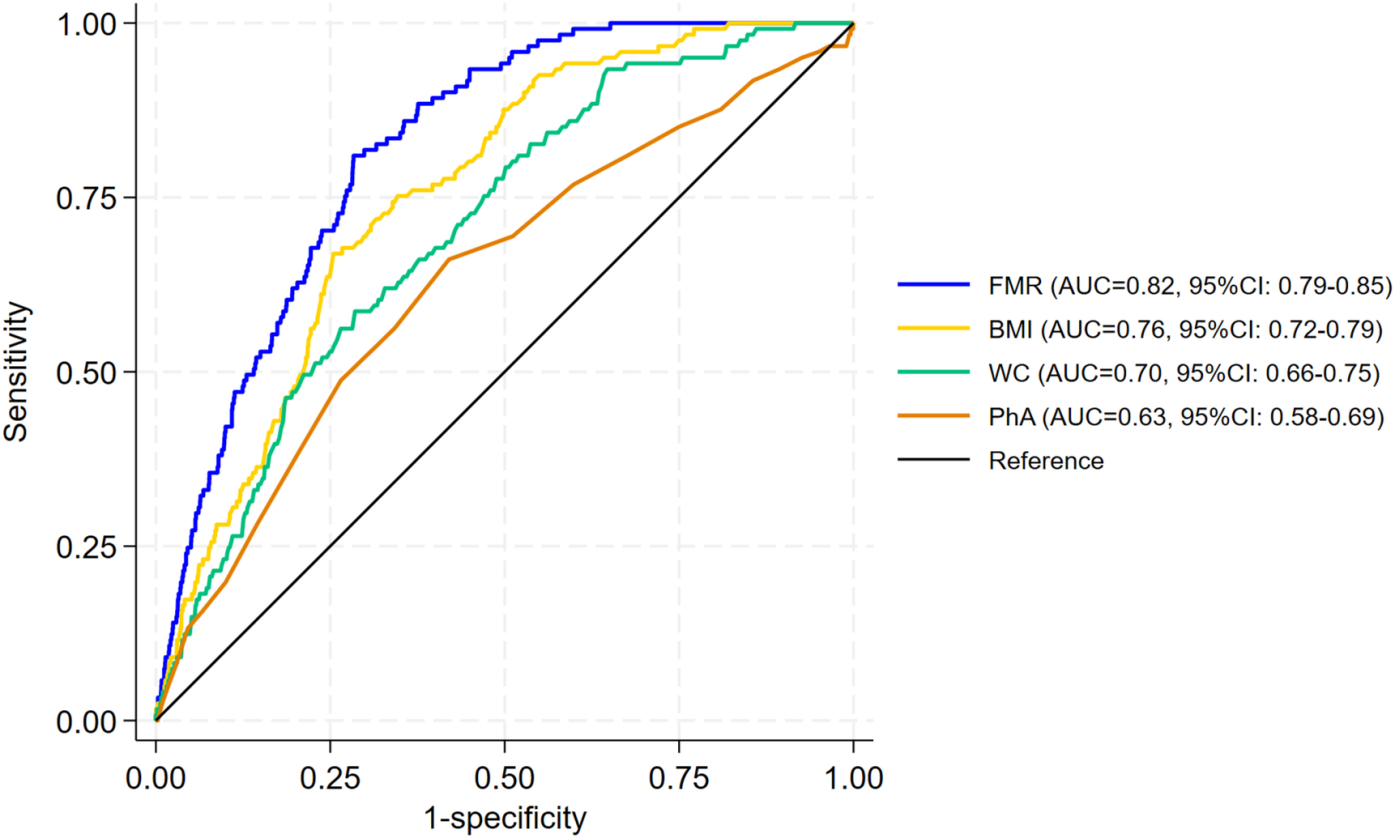
Receiver operating characteristic curves (ROC) for the screening tools to detect the risk of sarcopenic obesity in females.

**FIGURE 3:**
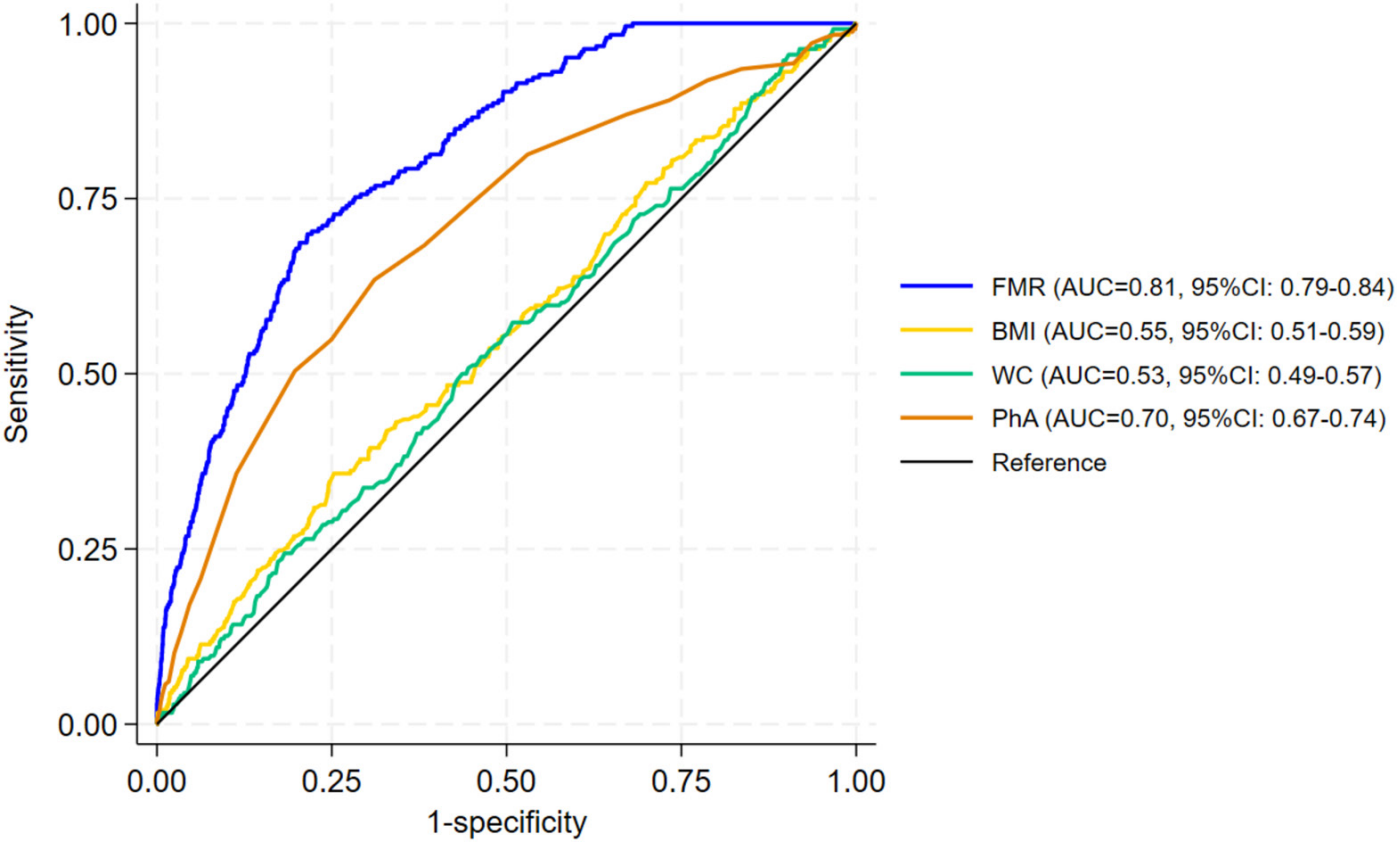
Receiver operating characteristic curves (ROC) for the screening tools used to detect the risk of sarcopenic obesity in males.

Figures 2 and 3 present the ROC curves illustrating the diagnostic performance of the screening tools for sarcopenic obesity. Among females, the AUCs for FMR, BMI, WC and PhA were 0.82 (95% CI: 0.79–0.85), 0.76 (95% CI: 0.72–0.79), 0.70 (95% CI: 0.66–0.75) and 0.63 (95% CI: 0.58–0.69), respectively. Among males, the corresponding AUCs were 0.81 (95% CI: 0.79–0.84), 0.55 (95% CI: 0.51–0.59), 0.53 (95% CI: 0.49–0.57) and 0.70 (95% CI: 0.67–0.74). According to the DeLong test, the AUC of FMR was significantly higher than those of BMI, WC and PhA in both sexes (all *p* < 0.001), confirming the superior discriminative performance of FMR for detecting sarcopenic obesity.

Table 2 summarizes the cutoff values, sensitivity, specificity, PPV and NPV of each screening tool for sarcopenic obesity. In females, FMR showed the highest sensitivity (81.0%) and relatively high specificity (71.6%) at a cutoff value of 0.602. In males, FMR also demonstrated strong diagnostic performance, with a sensitivity of 69.9% and specificity of 78.5% at a cutoff value of 0.388. By contrast, BMI and WC exhibited moderate diagnostic accuracy, whereas PhA demonstrated very high sensitivity but low specificity in both sexes. The PPVs were generally low across all indicators, reflecting the relatively low prevalence of sarcopenic obesity in the study population, whereas NPVs were consistently high, indicating the strong utility of these screening tools for ruling out sarcopenic obesity.

**TABLE 2:** Predictive ability of screening tools and cutoff values for sarcopenic obesity.

Screening tools	Cutoff	Sensitivity (%)	Specificity (%)	PPV (%)	NPV (%)
Fat to muscle ratio					
Female	0.602	81.0	71.6	7.0	99.3
Male	0.388	69.9	78.5	17.8	97.5
Body mass index					
Female	25	60.3	76.3	6.3	98.7
Male	25	35.8	72.4	7.9	94.4
Waist circumference					
Female	90	45.5	81.6	6.1	98.3
Male	85	59.8	43.0	6.5	94.1
Phase angle					
Female	5.93	96.7	0.8	2.5	90.7
Male	6.84	98.8	0.5	6.2	87.0

Abbreviations: NPV, negative predictive value; PPV, positive predictive value.

### 4.3 Association of the Screening Tools for Sarcopenic Obesity

The results of the univariate and multivariate logistic regression analyses are shown in Table 3. Most screening tools were significantly associated with sarcopenic obesity even after adjustment for age, education, physical inactivity, living arrangement, medication use, smoking, hypertension, heart disease, osteoporosis, respiratory disease, osteoarthritis, diabetes, GDS and MMSE. Among females, the ORs for sarcopenic obesity were FMR (OR: 3.06, 95% CI: 2.53–3.71), BMI (OR: 2.85, 95% CI: 2.35–3.47), WC (OR: 2.26, 95% CI: 1.86–2.74) and PhA (OR: 1.12, 95% CI: 0.93–1.34). Among males, the corresponding ORs were FMR (OR: 3.09, 95% CI: 2.67–3.58), BMI (OR: 1.43, 95% CI: 1.26–1.62), WC (OR: 1.24, 95% CI: 1.08–1.41) and PhA (OR: 0.65, 95% CI: 0.59–0.76).

**TABLE 3:** Associations between screening tools and sarcopenic obesity in multivariate logistic regression models: Overall population and subgroup aged < 75 years.

	Crude model			Adjusted model		
	OR	95% CI	*p* value	OR	95% CI	*p* value
Overall						
Fat‐to‐muscle ratio						
Female (per 1 SD)	2.44	2.10–2.84	< 0.001	3.06	2.53–3.71	< 0.001
Male (per 1 SD)	3.03	2.64–3.48	< 0.001	3.09	2.67–3.58	< 0.001
Body mass index						
Female (per 1 SD)	2.10	1.81–2.45	< 0.001	2.85	2.35–3.47	< 0.001
Male (per 1 SD)	1.26	1.12–1.41	< 0.001	1.43	1.26–1.62	< 0.001
Waist circumference						
Female (per 1 SD)	1.92	1.63–2.26	< 0.001	2.26	1.86–2.74	< 0.001
Male (per 1 SD)	1.13	0.99–1.28	0.061	1.24	1.08–1.41	0.002
Phase angle						
Female (per 1 SD)	0.66	0.54–0.80	< 0.001	1.12	0.93–1.34	0.225
Male (per 1 SD)	0.49	0.43–0.56	< 0.001	0.65	0.56–0.76	< 0.001
≤ 75 years						
Fat to muscle ratio						
Female (per 1 SD)	3.19	2.31–4.40	< 0.001	4.86	2.98–7.93	< 0.001
Male (per 1 SD)	3.49	2.78–4.37	< 0.001	3.62	2.81–4.66	< 0.001
Body mass index						
Female (per 1 SD)	3.12	2.25–4.34	< 0.001	4.56	2.81–7.41	< 0.001
Male (per 1 SD)	1.37	1.14–1.64	0.001	1.33	1.11–1.58	0.002
Waist circumference						
Female (per 1 SD)	2.94	2.05–4.20	< 0.001	3.53	2.20–5.67	< 0.001
Male (per 1 SD)	1.13	0.90–1.41	0.29	1.08	0.86–1.35	0.52
Phase angle						
Female (per 1 SD)	0.83	0.52–1.32	0.423	0.93	0.58–1.52	0.784
Male (per 1 SD)	0.52	0.42–0.66	0.001	0.50	0.39–0.64	< 0.001

*Note:* Crude model: Not adjusted for any variable. Adjusted model: Adjusted for age, education, physical inactivity, living alone, medication, smoking, hypertension, heart disease, osteoporosis, respiratory disease, osteoarthrosis, diabetes, GDS and MMSE.

Abbreviations: CI, confidence interval; GDS, geriatric depression scale; MMSE, minimental state examination; OR, odds ratio; SD, standard deviation.

### 4.4 Subgroup Analyses Limited to Participants Aged ≤ 75 Years

Results from the subgroup analysis of participants aged ≤ 75 years are presented in Table 3. Most screening tools remained significantly associated with sarcopenic obesity after multivariate adjustments for potential confounders. Among females, the ORs for sarcopenic obesity were FMR (OR: 4.86, 95% CI: 2.98–7.93), BMI (OR: 4.56, 95% CI: 2.81–7.41), WC (OR: 3.53, 95% CI: 2.20–5.67) and PhA (OR: 0.93, 95% CI: 0.58–1.52), with PhA showing no significant association. Among males, the ORs were FMR (OR: 3.62, 95% CI: 2.81–4.66), BMI (OR: 1.33, 95% CI: 1.11–1.58), WC (OR: 1.08, 95% CI: 0.86–1.35) and PhA (OR: 0.50, 95% CI: 0.39–0.64).

## 5 Discussion

This cross‐sectional study examined the associations between various screening tools for sarcopenic obesity in community‐dwelling older adults and demonstrated that FMR showed a stronger association with sarcopenic obesity, exhibiting higher diagnostic accuracy than BMI, WC and PhA.

The participant characteristics presented in Table 1 were consistent with previous findings [26], showing that individuals with sarcopenic obesity typically have lower bone mineral density [6, 27, 28] and a higher prevalence of osteoarthritis [29] and depression [5, 30] than those without. The relatively low prevalence of sarcopenic obesity in this study (female: 2.6%, male: 6.2%) aligns with earlier Japanese studies reporting rates between 1.7% and 8.2% [31, 32, 33, 34].

The strong association between FMR and sarcopenic obesity may be attributable to the fact that FMR reflects the key physiological features of the condition, reduced muscle mass, increased fat accumulation and diminished muscle strength [35]. Prior research has linked FMR not only to muscle function [36] but also to metabolic syndrome [37]. Because FMR captures the proportional balance between muscle and fat mass, it may serve as a practical and objective indicator for the early detection of sarcopenic obesity. Furthermore, as FMR can be measured using a single body composition device, it holds practical value for use in community and clinical settings, especially where assessment of VFA or grip strength is not feasible.

BMI was also associated with sarcopenic obesity, although its AUC was lower than that of FMR and notably weaker in males than in females. Because BMI does not distinguish between muscle and fat mass, it is a limited diagnostic tool for identifying sarcopenic obesity. Although BMI remains widely used as a convenient indicator of body composition, its interpretability varies across populations. For examples, obesity is defined as BMI ≥ 30 kg/m^2^ in most Western populations, but a lower threshold of ≥ 25 kg/m^2^ is typically applied Japan and other Asian cohorts due to elevated cardiometabolic risk at lower BMI values.

WC showed a significant association with sarcopenic obesity in females but a weaker association in males, which may reflect sex‐related differences in fat distribution, as central adiposity tends to be more pronounced in men regardless of sarcopenic status [38, 39, 40].

PhA was also associated with sarcopenic obesity, but its discriminative ability was lower than that of FMR. Although no studies have directly examined the relationship between sarcopenic obesity and PhA, previous research has demonstrated associations between PhA, sarcopenia and obesity. A systematic review reported that individuals with sarcopenia exhibit lower PhA values and that the prevalence of sarcopenia is higher among those with reduced PhA [15]. PhA is also known to decline in obesity due to increased adiposity, chronic inflammation and malnutrition [15]. Lower PhA values have been further associated with disability [41] and mortality [42], both of which are adverse clinical outcomes related to sarcopenic obesity.

Given that the diagnostic criteria for sarcopenic obesity in Japan apply to individuals aged ≤ 75 years, these findings offer important insights into the age‐specific validity of different screening tools. Notably, PhA was not significantly associated with sarcopenic obesity in females, regardless of age. This may reflect physiological differences between sexes, as PhA is influenced by cell membrane integrity and total body water distribution. Among females, hormonal variation and higher overall body fat percentage may reduce PhAs specificity as a marker of muscle quality.

Conversely, WC was significantly associated with sarcopenic obesity in males in the overall analysis, but this association was not observed among those aged ≤ 75 years. One possible explanation is that younger old men (≤ 75 years) may exhibit greater variability in visceral fat accumulation and muscle loss, diminishing WCs reliability as an indicator of sarcopenic obesity. Furthermore, because WC primarily reflects central adiposity, it may not fully capture the combined effects of reduced muscle mass and increased fat mass that characterize sarcopenic obesity.

The sensitivity, specificity, PPV and NPV of each screening tool for sarcopenic obesity are summarized in Table 2. FMR integrates measures of fat and muscle mass, directly reflecting the disproportion between these components that defines sarcopenic obesity. The high sensitivity and specificity observed in both sexes indicate that FMR may serve as a practical and informative index for community level screening. In contrast, BMI and WC, although widely used as general indicators of obesity, showed limited ability to distinguish sarcopenic obesity, likely because they do not account for differences in muscle mass. PhA demonstrated extremely high sensitivity but minimal specificity, suggesting that it may be affected by physiological factors unrelated to sarcopenic obesity, including hydration status and cellular integrity. Importantly, the overall low PPVs across all tools likely reflect the low prevalence of sarcopenic obesity (approximately 1%–2%) in this relatively healthy, community‐dwelling sample. However, the consistently high NPVs indicate that these tools, particularly FMR, are effective for ruling out sarcopenic obesity in older adults at the population level.

BIA is widely used in epidemiological research because of its simplicity, affordability and noninvasive nature. However, its accuracy can be affected by hydration status and the presence of oedema, potentially leading to misclassification of body composition [43]. Studies comparing BIA with DXA in hospitalized older adults [44] and individuals with metabolic syndrome [45] have shown that BIA tends to underestimate fat mass and overestimate muscle mass relative to DXA. Conversely, a systematic review in cancer patients found that BIA produced assessments comparable to DXA [46]. In our previous work, sarcopenic obesity diagnosed using BIA was significantly associated with the 5‐year incidence of long‐term care need [9]. Moreover, several previous studies published in high‐impact journals have successfully utilized BIA‐derived indices such as the fat‐to‐muscle ratio and muscle‐to‐fat ratio in to predict adverse health outcomes, supporting its value in large‐scale community‐based studies [17, 18, 47, 48].

This study has some limitations in generalizing its findings. First, due to its cross‐sectional design, causal relationships cannot be inferred, and the observed associations should be interpreted with caution. Second, the prevalence of sarcopenic obesity may have been underestimated because the NCGG‐SGS was conducted as a venue‐based health check‐up programme involving relatively healthy, community‐dwelling older adults. Third, as the data were collected at a single time point, temporal changes and the direction of causality could not be evaluated. Another limitation is that our findings were not validated against gold‐standard methods for body composition assessment, such as dual‐energy X‐ray absorptiometry (DXA) or magnetic resonance imaging. Future studies should conduct validation analysis using these reference methods to confirm the diagnostic performance of the indices evaluated in this study. Finally, the use of BIA to estimate body composition has inherent methodological constraints. Measurements obtained by BIA are influenced by hydration status and the presence of oedema, which may alter impedance values and bias the estimation of both muscle and fat mass. Such variability may have resulted in the misclassification of sarcopenia or obesity in this cohort, thereby affecting the estimated prevalence of sarcopenic obesity. Although BIA remains widely used in large‐scale epidemiological research due to its accessibility, feasibility and low cost, these limitations should be considered when interpreting the results.

## 6 Conclusions

In community‐dwelling older adults, FMR is significantly associated with sarcopenic obesity and appears to be a more practical and informative screening tool than BMI and PhA. However, whether FMR can predict the future development of sarcopenic obesity should be clarified through longitudinal studies.

## Funding

This work was supported by the Health and Labour Sciences Research Grant from the Japanese Ministry of Health, Labour and Welfare (H23‐tyoujyu‐ippan‐001 and H24‐tyoujyu‐ippan‐004); the Research Funding for Longevity Sciences from the National Center for Geriatrics and Gerontology (22‐16, 24‐18 and 25‐26); Obu City Local Government; the Grant‐in‐Aid for Scientific Research (B) from the Japan Society for the Promotion of Science (23300205); the Strategic Basic Research Programs (RISTEX Redesigning Communities for Aged Society) from the Japan Science and Technology Agency; and the Japan Agency for Medical Research and Development (AMED) under Grant Numbers 15dk0107003h0003 and 15dk0207004h0203. The funders had no role in the design or conduct of the study, the collection, management, analysis or interpretation of data, or the preparation, peer review or approval of the manuscript.

## Ethics Statement

The Ethics Committee of the National Center for Geriatrics and Gerontology (NCGG) approved the study protocol (Registration Number 1440‐8). The study was conducted in accordance with the ethical standards laid down in the 1964 Declaration of Helsinki and its later amendments. Written informed consent was obtained from all participants prior to their inclusion in the study.

## Conflicts of Interest

The authors declare no conflicts of interest.
